# A Fast Approach to Bimatrix Games with Intuitionistic Fuzzy Payoffs

**DOI:** 10.1155/2014/121245

**Published:** 2014-08-14

**Authors:** Min Fan, Ping Zou, Shao-Rong Li, Chin-Chia Wu

**Affiliations:** ^1^Faculty of Science, Kunming University of Science and Technology, Kunming 650500, China; ^2^Faculty of Management and Economic, Kunming University of Science and Technology, Kunming 650500, China; ^3^Faculty of Economic, Peking University, Beijing 100083, China; ^4^Department of Statistics, Feng Chia University, Taichung 407, Taiwan

## Abstract

The aim of this paper is to develop an effective method for solving bimatrix games with payoffs of intuitionistic fuzzy value. Firstly, bimatrix game model with intuitionistic fuzzy payoffs (IFPBiG) was put forward. Secondly, two kinds of nonlinear programming algorithms were discussed with the Nash equilibrium of IFPBiG. Thirdly, Nash equilibrium of the algorithm was proved by the fixed point theory and the algorithm was simplified by linear programming methods. Finally, an example was solved through Matlab; it showed the validity, applicability, and superiority.

## 1. Introduction

Since the 1940s, game theory [1, 2] has been developed to descript, analyze, and solve the duels among a group of rational agents with strategical behavior. Among the game theory, matrix games have been extensively studied [3–23] and successfully applied to some fields [24–27]. It is becoming an important research field which can be classified into cooperative games and noncooperative games, zero sum games and nonzero sum games (bimatrix games), and crisp matrix games and fuzzy matrix games. In this paper, we mainly deal with fuzzy bimatrix game, one of the most important types of noncooperative games.

In real game situations, due to a lack of information or imprecision of the available information, players could only estimate the payoff value approximately with some imprecise degree. The fuzzy set [28] uses only a membership function to indicate a degree of belongingness to the fuzzy set under consideration. A degree of nonbelongingness is just automatically the complement to one. In 1986, Atanassov [29] introduced the concept of intuitionistic fuzzy sets (IF-sets), which is characterized by two functions expressing the degree of belongingness and the degree of nonbelongingness, respectively. The idea of the IF-sets made the description more close to the actual situation in fuzzy matrix games.

Matrix games with mathematical programming are a mainstream research direction [9–23], also the fuzzy matrix games and the bimatrix games. Recently, the studies of intuitionistic fuzzy matrix games have successfully applied to more fields, such as marketing, finance, and advertising. In theoretical research [9–11], Larbani, 2009, solved bimatrix games with fuzzy payoffs by introducing nature as a third player. Li and his team get a lot of research results [15–20] in intuitionistic fuzzy matrix games, such as intuitionistic fuzzy objective, biobjective, payoffs of triangular intuitionistic fuzzy numbers, payoffs of interval-valued fuzzy numbers, payoffs of interval-valued intuitionistic fuzzy numbers and so forth.

However, fuzzy bimatrix games with intuitionistic fuzzy payoffs (IFPBiG) are less studied, although in the real game problems, IFPBiG is very common. For example, in the research about market share games between real estate enterprises, the expectations of market share were very difficult to accurately estimate under the complicated situation, but fuzzy language could be used to express the satisfaction degree and rejection degree of the market share. For example, under the one of the situation, player I has the payoff value as (0.7 and 0.1) which means that for player I, the satisfaction degree is 0.7, the rejection degree is 0.1, and the hesitation degree is 0.2. It could be made clear by voting model that there are 70% of people voted satisfied, 10% of people voted against, and 20% of people abstained from voting. Due to the incompleteness and uncertainty of the market information, the payoff value of players I and II is not necessarily a zero-sum, and this kind of phenomenon was very common.

The focus of this paper is considering the effective method for solving IFPBiG problem, with the Nash equilibrium being proved by the fixed point theory.

This paper is arranged as follows.  Section 2 briefly reviews some concepts such as IF-sets, IFPBiG, and solution concepts of IFPBiG.  Section 3 obtains linear programming methods for IFPBiG with the transforms. In Section 4, the proposed method was illustrated with a numerical example and showed the validity and applicability. Conclusion was made in Section 5.

## 2. Preliminaries

In this section, some basic definitions and operations of intuitionistic fuzzy sets and game theory were briefly reviewed, which are used in the following sections.

### 2.1. The Concept and Operations of IF-Sets

**Definition 1 (Atanassov [29]).**

Let

(1) A=〈x,μA(x),vA(x)〉,   ∀x∈U,  0≤μA(x)+vA(x)≤1,B=〈x,μB(x),vB(x)〉, ∀x∈U,  0≤μB(x)+vB(x)≤1

be two IF-sets of *U*. Then they have the following properties.

1. $\;\bar{A}=\langle x,v_{A}\left(x\right),\mu_{A}(x)\rangle$.
2. *A*⊆*B*⇔*μ*_*A*_(*x*) ≤ *μ*_*B*_(*x*), *v*_*A*_(*x*) ≤ *v*_*B*_(*x*).
3. *A* = *B*⇔*A*⊆*B*∧*B*⊆*A*.
4. *A*∩*B* = 〈*X*, *μ*_*A*_(*x*)∧*μ*_*B*_(*x*), *v*_*A*_(*x*)∨*v*_*B*_(*x*)〉.
5. *A* ∪ *B* = 〈*X*, *μ*_*A*_(*x*)∨*μ*_*B*_(*x*), *v*_*A*_(*x*)∧*v*_*B*_(*x*)〉.
6. *A* + *B* = 〈*X*, *μ*_*A*_(*x*) + *μ*_*B*_(*x*) − *μ*_*A*_(*x*)*μ*_*B*_(*x*), *v*_*A*_(*x*)*v*_*B*_(*x*)〉.
7. *AB* = 〈*X*, *μ*_*A*_(*x*)*μ*_*B*_(*x*), *v*_*A*_(*x*) + *v*_*B*_(*x*) − *v*_*A*_(*x*)*v*_*B*_(*x*)〉.
8. *λA* = 〈*x*, 1 − (1 − *μ*_*A*_(*x*))^*λ*^, (*v*_*A*_(*x*))^*λ*^〉.
9. *A* ^*λ*^ = 〈*x*, (*μ*_*A*_(*x*))^*λ*^, 1 − (1 − *v*_*A*_(*x*))^*λ*^〉.

### 2.2. IFPBiG and Solution Concepts

Let *S*_1_ and *S*_2_ be sets of pure strategies for players I and II, respectively;

(2) αi∈S1 (i∈I,I={1,2,…,m})βj∈S2 (j∈J,J={1,2,…,n}).

*X* and *Y* are known as the mixed strategies for players I and II, respectively. Consider

(3) X={xi∈Rm,∑i=1mxi=1, xi≥0, i=1,2,…,m},Y={yi∈Rn,∑j=1nyj=1, yj≥0, j=1,2,…,n},

where *x*_*i*_ (*i* = 1, 2,…, *m*) and *y*_*j*_  (*j* = 1, 2,…, *n*) are probabilities in which players I and II choose their pure strategies.

At the outcome (*α*_*i*_, *β*_*j*_), the payoffs of players I and II are represented as IF-sets 〈*μ*_*ij*_^*A*^, *ν*_*ij*_^*A*^〉,  〈*μ*_*ij*_^*B*^, *ν*_*ij*_^*B*^〉. Thus IFPBiG is concisely expressed in the intuitionistic fuzzy matrix form as follows:

(4) A~=(〈μijA,νijA〉)m×n,B~=(〈μijB,νijB〉)m×n.

If players I and II choose *X* and *Y* as mixed strategies, respectively, according to (7) and (8) of properties followed Definition 1, the expected payoff of player I can be calculated as follows:

(5) EA(x,y)=xTA~y=(x1,x2,…,xm) ×(〈μ11A,ν11A〉〈μ12A,ν12A〉⋯〈μ1nA,ν1nA〉〈μ21A,ν21A〉〈μ22A,ν22A〉⋯〈μ2nA,ν2nA〉⋯⋯⋯〈μm1A,νm1A〉〈μm2A,νm2A〉⋯〈μmnA,νmnA〉) ×(y1y2⋮yn)=(〈1−∏i=1m∏j=1n(1−μijA)xiyj,∏i=1m∏j=1n(νijA)xiyj〉)m×n=Δ(μEA,νEA).

In the same way, the expected payoff of player II can be calculated as follows:

(6) EB(x,y)=xTB~y=(〈1−∏i=1m∏j=1n(1−μijB)xiyj,∏i=1m∏j=1n(νijB)xiyj〉)m×n=Δ(μEB,νEB).

**Definition 2 .**

IFPBiG may be expressed as $G_{\text{IF}}^{P}=(X,Y;\tilde{A},\tilde{B};S_{1},S_{2})$.

**Definition 3 .**

(x*, y*) ∈ (*X* × *Y*) is called a reasonable solution if and only if

(7) $$\begin{array}{c} x^{*T}\tilde{A}y^{*}\geq x^{T}\tilde{A}y^{*},\\ x^{*T}\tilde{A}y^{*}\geq x^{*T}\tilde{B}y. \end{array}$$

Combining with Definition 1 and Definition 3, it gets the following theorem.

**Theorem 4 .**

(x*, y*) ∈ (*X* × *Y*) is called a reasonable solution if and only if

(8) $$\begin{array}{c} \exists \left(x^{*},y^{*}\right)\{\begin{array}{c} \mu_{EA}\left(x^{*},y^{*}\right)\geq \mu_{EA}\left(x_{i},y_{j}\right)\\ \nu_{EA}\left(x^{*},y^{*}\right)\leq \nu_{EA}\left(x_{i},y_{j}\right)\\ \mu_{EB}\left(x^{*},y^{*}\right)\geq \mu_{EB}\left(x_{i},y_{j}\right)\\ \nu_{EB}\left(x^{*},y^{*}\right)\leq \nu_{EB}\left(x_{i},y_{j}\right). \end{array} \end{array}$$

**Proof**

(1) Consider

(9) ∀y∈Y,  let  X(y)={x∗∈X,μEA(x∗,y∗)≥μEA(xi,yj), νEA(x∗,y∗)≤νEA(xi,yj)};

then *X*(*y*) ⊂ *X* and *X*(*y*) are the mixed strategies which make the expected satisfaction degree of player I be maximum and the expected reject degree be minimum of player II, when player II use the mixed strategies *y*.

In the same way,

(10) ∀x∈X,Y(x)={y∗∈Y,μEB(x∗,y∗)≥μEB(xi,yj), νEB(x∗,y∗)≤νEB(xi,yj)},

where *Y*(*x*) ⊂ *Y* and *Y*(*x*) are the mixed strategies which make the expected satisfaction degree of player II be maximum and the expected reject degree be minimum of player I, when player I uses the mixed strategies *x*.

From the properties of Definition 1, it gets

(11) ∀x1∗,x2∗∈X(y)⊂X, λ1x1∗+(1−λ1)x2∗∈XμEA(λ1x1∗+(1−λ1)x2∗,y) =μEA(λ1x1∗,y)+μEA((1−λ1)x2∗,y) =λ1μEA(x1∗,y)+(1−λ1)μEA(x2∗,y)νEA(λ1x1∗+(1−λ1)x2∗,y)  =νEA(λ1x1∗,y)+νEA((1−λ1)x2∗,y)  =λ1νEA(x1∗,y)+(1−λ1)νEA(x2∗,y)∀x1∗,x2∗∈X(y)⊂X,λ∈[0,1]λ μEA(x1∗,y)≥λμEA(x,y)(1−λ)μEB(x2∗,y)≥(1−λ)μEB(x,y)λνEA(x1∗,y)≤λνEA(x,y)(1−λ)νEB(x2∗,y)≤(1−λ)νEB(x,y).

Then,

(12) $$\begin{array}{c} \mu_{EA}\left(\lambda x_{1}^{*}+\left(1-\lambda \right)x_{2}^{*},y\right)\geq \mu_{EA}\left(x,y\right)\\ \nu_{EA}\left(\lambda x_{1}^{*}+\left(1-\lambda \right)x_{2}^{*},y\right)\leq \nu_{EA}\left(x,y\right)\\ \lambda x_{1}^{*}+\left(1-\lambda \right)x_{2}^{*}\in X\left(y\right), \end{array}$$

so *X*(*y*) is a convex set and *Y*(*x*) is also a convex set.

(2) Consider *F* : *X* × *Y* → *P*(*X* × *Y*),  *F*(*z*) = *X*(*y*) × *Y*(*x*),  ∀*z* = (*x*, *y*) ∈ (*X* × *Y*),

(13) $$\begin{array}{c} \left(\psi ,\varphi \right)\in F\left(z\right)⟺\psi \in X\left(y\right),\;\;\varphi \in Y\left(x\right). \end{array}$$

 Let *z*_*n*_ = (*x*_*n*_, *y*_*n*_)^*T*^ and (*ψ*_*n*_, *φ*_*n*_) ∈ *F*(*z*_*n*_)
 when *n* → +*∞*,  *ψ*_*n*_ → *ψ*_0_, *φ*_*n*_ → *φ*_0_, *x*_*n*_ → *x*_0_, *y*_*n*_ → *y*_0_
 as (*ψ*_*n*_, *φ*_*n*_) ∈ *F*(*z*_*n*_) so *ψ*_*n*_ ∈ *X*(*y*_*n*_)
  (14) $$\begin{array}{c} \forall x\in X,\;\mu \left(\psi_{n}^{T}\tilde{A}y\right)\geq \mu \left(x^{T}\tilde{A}y_{n}\right). \end{array}$$
 So $\lim_{n\to \infty }\mu (\psi_{n}^{T}\tilde{A}y)\geq \lim_{n\to \infty }\mu (x^{T}\tilde{A}y_{n})$
 as $\lim_{n\to \infty }\mu (x_{n}^{T}\tilde{A}y_{n})=\mu (x_{0}^{T}\tilde{A}y_{0})$ and so $\mu (\psi_{0}^{T}\tilde{A}y_{0})\geq \mu (x^{T}\tilde{A}y_{0})$ that means *ψ*_0_ ∈ *X*(*y*_0_).
 Same as *φ*_0_ ∈ *Y*(*x*_0_), then *X*(*y*), *Y*(*x*) are all convex sets, so *F*(*z*) is convex set too.
 As (*ψ*, *φ*) ∈ *F*(*z*)⇔*ψ* ∈ *X*(*y*), *φ* ∈ *Y*(*x*)
 so (*ψ*_0_, *φ*_0_) ∈ *F*(*z*_0_).
 
*F* is the upper continuous; meanwhile, set-valued mapping *F* exists fixed point.
 There is a point; make z* ∈ *F*(z*)
  (15) x∗TA~y∗≥xTA~y∗x∗TA~y∗≥x∗TB~y.

The proof is completed.

## 3. Linear Programming Methods for IFPBiG

From the above, the mixed Nash equilibrium solution of IFPBiG can be obtained by solving the following programming problems:

(16) max⁡{μ1−ν1}1−∏i=1m(1−μijA)xi≥μ1∏i=1m(νijA)xi≤ν1∑i=1mxi=10≤μ1+ν1≤1xi≥0, μ1≥0, ν1≥0,max⁡{μ2−ν2}1−∏j=1n(1−μijB)Yj≥μ2∏j=1n(νijB)Yj≤ν2∑j=1nyj=10≤μ2+ν2≤1yj≥0, μ2≥0, ν2≥0.

The above two nonlinear programming models can be transformed into the following linear programming model by “linear exchange”:

(17) max⁡{μ1−ν1+μ2−ν2}λ1∑i=1mxiln⁡⁡(1−μijA)+(1−λ1)∑i=1mxiln⁡νijA ≤λ1ln⁡μ1+(1−λ1)ln⁡ν1λ2∑j=1nyjln⁡⁡(1−μijB)+(1−λ2)∑j=1nyjln⁡νijB ≤λ2ln⁡μ2+(1−λ2)ln⁡ν2∑i=1mxi=1∑j=1nyj=10≤μ1+ν1≤1, 0≤μ2+ν2≤1xi≥0, μ1≥0, ν1≥0, 0≤λ1≤1yj≥0, μ2≥0, ν2≥0, 0≤λ2≤1,

where *λ*_1_, *λ*_2_ are “optimistic coefficient” of players I and II, respectively, which is standard for the rationality of players.

## 4. Numerical Example

To test our algorithm above, the following experiment was made.

There were two major hydropower enterprises competed for the power supply qualification through bidding. Both sides can take the fact that the bidding prices strategies are “high, flat, and low.” And both sides made up a think-tank to vote about the satisfaction degree and reject degree of each situation. The data of corresponding enterprises *A* and *B* is

(18) $$\begin{array}{c} \tilde{A}=\begin{array}{ccc} \begin{array}{c} \text{high}\\ \text{flat}\\ \text{low} \end{array} & \left(\begin{array}{ccc} \langle 0.95,0.05\rangle & \langle 0.7,0.25\rangle & \langle 0.5,0.4\rangle \\ \langle 0.7,0.25\rangle & \langle 0.7,0.25\rangle & \langle 0.25,0.7\rangle \\ \langle 0.25,0.7\rangle & \langle 0.5,0.4\rangle & \langle 0.05,0.95\rangle \end{array}\right) & \\ & \begin{array}{ccc} \text{high} & \;\;\text{flat} & \;\;\;\text{low} \end{array} & \end{array}\\ \tilde{B}=\begin{array}{ccc} \begin{array}{c} \text{high}\\ \text{flat}\\ \text{low} \end{array} & \left(\begin{array}{ccc} \langle 0.5,0.4\rangle & \langle 0.25,0.7\rangle & \langle 0.05,0.95\rangle \\ \langle 0.5,0.4\rangle & \langle 0.7,0.25\rangle & \langle 0.5,0.4\rangle \\ \langle 0.7,0.25\rangle & \langle 0.7,0.25\rangle & \langle 0.5,0.4\rangle \end{array}\right) & \\ & \begin{array}{ccc} \text{high} & \;\;\text{flat} & \;\;\;\text{low} \end{array} & \end{array} \end{array}$$

where *a*_12_ = 〈0.7,0.25〉 means that if enterprise *A* chooses high price, enterprise *B* chooses flat price, with 70% of experts believe that the expected profit is satisfied, 25% of experts argue that it is not satisfied, and 5% of the experts cannot judge. The other elements in the matrix can be also explained like this:

“optimistic coefficients” *λ*_1_, *λ*_2_ of different players have been taking several common situations in this example. Then it gets the following linear programming model:

(19) max⁡{μ1−v1+μ2−v2},λ1(x1ln⁡⁡0.05+x2ln⁡⁡0.3+x3ln⁡⁡0.75)+(1−λ1) ×(x1ln⁡⁡0.05+x2ln⁡⁡0.25+x3ln⁡⁡0.7) −[λ1ln⁡⁡μ1+(1−λ1)ln⁡⁡v1]≤0,λ1(x1ln⁡⁡0.5+x2ln⁡⁡0.3+x3ln⁡⁡0.5)+(1−λ1) ×(x1ln⁡⁡0.25+x2ln⁡⁡0.25+x3ln⁡⁡0.4) −[λ1ln⁡⁡μ1+(1−λ1)ln⁡⁡v1]≤0,λ1(x1ln⁡⁡0.5+x2ln⁡⁡0.75+x3ln⁡⁡0.95)+(1−λ1) ×(x1ln⁡⁡0.4+x2ln⁡⁡0.7+x3ln⁡⁡0.95) −[λ1ln⁡⁡μ1+(1−λ1)ln⁡⁡v1]≤0,λ2(y1ln⁡⁡0.5+y2ln⁡⁡0.5+y3ln⁡⁡0.3)+(1−λ2) ×(y1ln⁡⁡0.4+y2ln⁡⁡0.4+y3ln⁡⁡0.25) −[λ2ln⁡⁡μ2+(1−λ2)ln⁡⁡v2]≤0,λ2(y1ln⁡⁡0.75+y2ln⁡⁡0.3+y3ln⁡⁡0.3)+(1−λ2) ×(y1ln⁡⁡0.7+y2ln⁡⁡0.25+y3ln⁡⁡0.25) −[λ2ln⁡⁡μ2+(1−λ2)ln⁡⁡v2]≤0,λ2(y1ln⁡⁡0.95+y2ln⁡⁡0.5+y3ln⁡⁡0.5)+(1−λ2) ×(y1ln⁡⁡0.95+y2ln⁡⁡0.4+y3ln⁡⁡0.4) −[λ2ln⁡⁡μ2+(1−λ2)ln⁡⁡v2]≤0,∑i=1mxi=1∑j=1myj=10≤μ1+v1≤1,  0≤μ2+v2≤1xi≥0,  μ1≥0,  v1≥0,  0≤λ1≤1yj≥0,  μ2≥0,  v2≥0,  0≤λ2≤1.

Matlab 7.0 was used to solve the above nonlinear programming and the results were as shown in Table 1.

### 4.1. The Solution and Expectation of Satisfaction Degree and Reject Degree

The first two columns were the optimistic coefficient of enterprises *A* and *B*, respectively. Column *x* and *y* were mixed strategies, respectively. The last two columns were total expectations, respectively; they were shown as the intuitionistic fuzzy sets and they stand for the satisfaction degree and reject degree.

(1) Consider *λ*_1_ = *λ*_2_ = 0.5: it means that the satisfaction degree and reject degree of both enterprises are equal. It stands for a moderate rationality and it is the most common case.

At this time, enterprise *A* will take the “high” strategy; its probability is 0.41, and enterprise *B* will take “flat” strategy, the probability of 0.57. The satisfaction degree and reject degree of forecast expected profit for *A* and *B* are (0.67 and 0.31) and (0.51 and 0.42), respectively.

(2) Consider *λ*_1_ = *λ*_2_ = 0.1: it means that both enterprises are very careful about reject degree; it stands for a pessimistic rationality.

At this time, enterprise *A* will take the “flat” strategy; its probability is 0.53, and enterprise *B* will take “high” strategy, the probability of 0.38. The satisfaction degree and reject degree of forecast expected profit for *A* and *B* are (0.56 and 0.41) and (0.48 and 0.43), respectively, and they both declined compared with Case (1).

(3) Consider *λ*_1_ = *λ*_2_ = 0.9: it means that both enterprises are very careful about satisfaction degree; it stands for an optimistic rationality.

At this time, enterprise *A* will take the “flat” strategy; its probability is 0.49, and enterprise *B* will take “flat” strategy, the probability of 0.42. The satisfaction degree and reject degree of forecast expected profit for enterprises *A* and *B* are (0.75 and 0.17) and (0.78 and 0.20), respectively, and they both increase significantly compared with Case (1); it looks like a win-win situation.

(4) Consider *λ*_1_ = 0.1  *λ*_2_ = 0.9 or *λ*_1_ = 0.9  *λ*_2_ = 0.1: it means that both enterprises meet each other with the extreme rationality.

From Table 1, the optimal solution is *λ*_1_ = 0.9  *λ*_2_ = 0.9; both enterprises take “flat” strategy; they could get maximum expected profit of 0.75 and 0.78, respectively.

## 5. Conclusion

1. Under the complicated decision environment, the model and method of this paper were more simple and practical than other general equilibrium game model because of the “linear exchange.” Meanwhile “optimistic coefficients” *λ*_1_, *λ*_2_ of different players also can fully express the opinions of the experts, as well as the rationality of decision makers.
2. Further study can be focused on more people's bidding online intuitionistic fuzzy matrix game model, dynamic intuitionistic fuzzy bimatrix game model, more people cooperation game model, and so on.

## Figures and Tables

**Table 1:** The solution and expectation of satisfaction degree and reject degree.

*λ*_1_	*λ*_2_	*x*	*y*	*XA* *Y*	*XB* *Y*
0.5	0.5	(0.41, 0.31, 0.28)	(0.18, 0.57, 0.25)	(0.67, 0.31)	(0.51, 0.42)
0.1	0.1	(0.11, 0.53, 0.36)	(0.38, 0.34, 0.28)	(0.56, 0.41)	(0.48, 0.43)
0.9	0.9	(0.21, 0.49, 0.30)	(0.28, 0.42, 0.3)	(0.75, 0.17)	(0.78, 0.20)
0.1	0.9	(0.22, 0.31, 0.47)	(0.31, 0.43, 0.26)	(0.40, 0.41)	(0.52, 0.33)
0.9	0.1	(0.61, 0.14, 0.25)	(0.17, 0.35, 0.48)	(0.49, 0.41)	(0.48, 0.45)
